# The impact of pyrethroid-pyriproxyfen and pyrethroid-chlorfenapyr long-lasting insecticidal nets on density of primary malaria vectors *Anopheles gambiae* s.s. and *Anopheles coluzzii* in Benin: a secondary analysis of a cluster randomised controlled trial

**DOI:** 10.1186/s13071-023-06104-5

**Published:** 2024-01-04

**Authors:** Boulais Yovogan, Arthur Sovi, Armel Djènontin, Constantin J. Adoha, Bruno Akinro, Manfred Accrombessi, Edouard Dangbénon, Come Z. Koukpo, Zul-Kifl Affolabi, Prudenciène A. Agboho, Casimir Dossou Kpanou, Landry Assongba, Antoine Abel Missihoun, Tatchémè Filémon Tokponnon, Clément Agbangla, Germain Gil Padonou, Louisa A. Messenger, Corine Ngufor, Jackie Cook, Martin C. Akogbéto, Natacha Protopopoff

**Affiliations:** 1 Faculté des Sciences et Techniques, Université d’Abomey-Calavi, Abomey-Calavi, Benin; 2 Centre de Recherche Entomologique de Cotonou, Cotonou, Benin; 3 Faculté d’Agronomie, Université de Parakou, Parakou, Benin; 4 Faculty of Infectious and Tropical Diseases, Department of Disease Control, The London School of Hygiene and Tropical Medicine, London, UK; 5 Ecole Polytechnique d’Abomey-Calavi, Université d’Abomey-Calavi, Abomey-Calavi, Benin; 6 Department of Environmental and Occupational Health, School of Public Health, University of Nevada, Las Vegas, NV 89154 USA; 7 Medical Research Council (MRC) International Statistics and Epidemiology, Epidemiology Group, London School of Hygiene and Tropical Medicine, London, UK

**Keywords:** Dual active-ingredients LLINs, Density, *Anopheles coluzzii*, *Anopheles gambiae* s.s., Benin

## Abstract

**Background:**

Long-lasting insecticidal nets (LLINs) may have different impacts on distinct mosquito vector species. We assessed the efficacy of pyrethroid-pyriproxyfen and pyrethroid-chlorfenapyr LLINs on the density of *Anopheles gambiae* s.s. and *An. coluzzii* compared to pyrethroid-only nets in a three-arm cluster randomised control trial in Benin*.*

**Methods:**

Indoor and outdoor collections of adult mosquitoes took place in 60 clusters using human landing catches at baseline and every 3 months for 2 years. After morphological identification, around 15% of randomly selected samples of *An. gambiae* s.l. were dissected to determine parity, species (using PCR).

**Results:**

Overall, a total of 46,613 mosquito specimens were collected at baseline and 259,250 in the eight quarterly collections post-net distribution. Post-net distribution, approximately 70% of the specimens of *An. gambiae* s.l. speciated were *An. coluzzii*, while the rest were mostly composed of *An. gambiae* s.s. with a small proportion (< 1%) of hybrids (*An. gambiae/coluzzii*). There was no evidence of a significant reduction in vector density indoors in either primary vector species [*An. coluzzii*: DR (density ratio) = 0.62 (95% CI 0.21–1.77), *p* = 0.3683 for the pyrethroid-pyriproxyfen LLIN and DR = 0.56 (95% CI 0.19–1.62), *p* = 0.2866 for the pyrethroid-chlorfenapyr LLIN, *An. gambiae* s.s.: DR = 0.52 (95% CI 0.18–1.46), *p* = 0.2192 for the pyrethroid-pyriproxyfen LLIN and DR = 0.53 (95% CI 0.19–1.46), *p* = 0.2222 for the pyrethroid-chlorfenapyr]. The same trend was observed outdoors. Parity rates of *An. gambiae* s.l. were also similar across study arms.

**Conclusions:**

Compared with pyrethroid-only LLINs, pyrethroid-chlorfenapyr LLINs and pyrethroid-pyriproxyfen LLINs performed similarly against the two primary mosquito species *An. gambiae* s.s. and *An. coluzzii* in Benin.

**Graphical Abstract:**

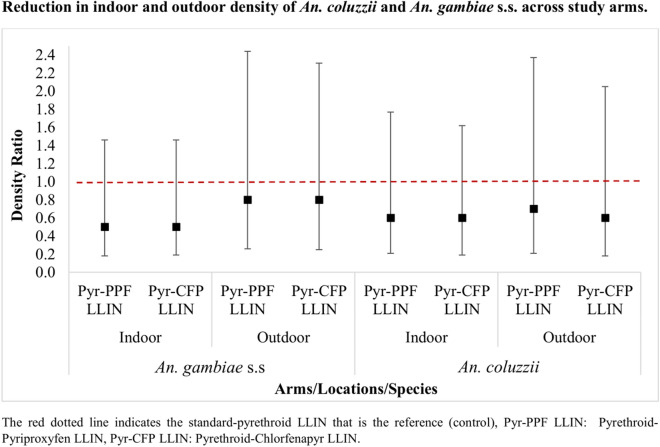

**Supplementary Information:**

The online version contains supplementary material available at 10.1186/s13071-023-06104-5.

## Background

After 2 decades of success in reducing the malaria burden in sub-Saharan Africa, cases are now increasing in many countries [1]. Some of the factors explaining this resurgence are widespread pyrethroid resistance in *Anopheles* vectors of malaria and more recently the disruptions caused by the COVID-19 pandemic [1]. Given that pyrethroid-only long-lasting insecticidal nets (LLINs) were the sole class of nets recommended for community use by the World Health Organization (WHO) until recently, and the increasingly worrying epidemiological situation of malaria globally, urgent actions aiming to develop a new generation of LLINs are needed.

LLINs incorporating a mixture of a pyrethroid insecticide plus piperonyl butoxide demonstrated better efficacy on malaria than standard pyrethroid-only LLINs [2, 3] and were the first new second-generation LLINs to receive a WHO policy recommendation in 2017 [4]. Two other types of LLIN incorporating a pyrethroid and a second insecticide with a different mode of action, either pyriproxyfen (an insect growth regulator that inhibits fertility) or chlorfenapyr (a pyrrole insecticide which disrupts mitochondrial oxidative phosphorylation), were assessed in randomized controlled trials (RCTs) in Tanzania [5] and Benin [6]. In both trials, Interceptor G2^®^ LLINs (mixture of alpha-cypermethrin-chlorfenapyr) provided clear additional protection against malaria compared to standard LLINs with 44 and 46% reductions in malaria case incidence after 2 years of follow-up in Tanzania and Benin, respectively. The effect of Royal Guard^®^ LLINs (mixture of alphacypermethrin-pyriproxyfen) was not as evident and reductions in malaria incidence was marginal in both countries [5, 6]. In March 2023, Interceptor^®^ G2, the first pyrethroid-chlorfenapyr LLIN in class product, received a full recommendation from the WHO, while the pyrethroid-pyriproxyfen LLIN, Royal Guard^®^, was given a conditional recommendation pending additional evidence on efficacy [7].

Entomological indicators play a crucial role in understanding epidemiological results, as the impact of vector control interventions may vary depending on vector species composition, behaviours (outside/inside biting or resting) and insecticide resistance [8]. Some insecticides may be more effective on secondary vectors rather than primary ones in an area; a better understanding of these phenomena will help refine future prevention strategies. In the Tanzania RCT, the pyrethroid-chlorfenapyr LLINs were the most effective against *Anopheles funestus* s.l. for 3 years, with PBO LLINs remaining effective for 2 years. The same authors also showed that neither of the dual active-ingredient (ai) LLINs succeeded in controlling *Anopheles arabiensis* [9]. The main entomological outcomes of the trial in Benin were reported for three malaria vector complexes (*Anopheles gambiae* s.l., *An. funestus* and *An. nili*) pooled together [6]. In Benin, the two primary vectors are *Anopheles gambiae* s.s. and *An. coluzzii* (both part of the *An. gambiae* s.l complex) with composition and insecticide resistance frequencies varying across the country [10, 11].

The present study reports a secondary analysis of the RCT entomological data investigating the efficacy of Royal Guard^®^ LLINs and Interceptor^®^ G2 LLINs compared to pyrethroid-only LLINs on the two primary vectors found in the study area, *An. coluzzii* and *An. gambiae* s.s.

## Methods

### Study area and design

**Fig. 1:**
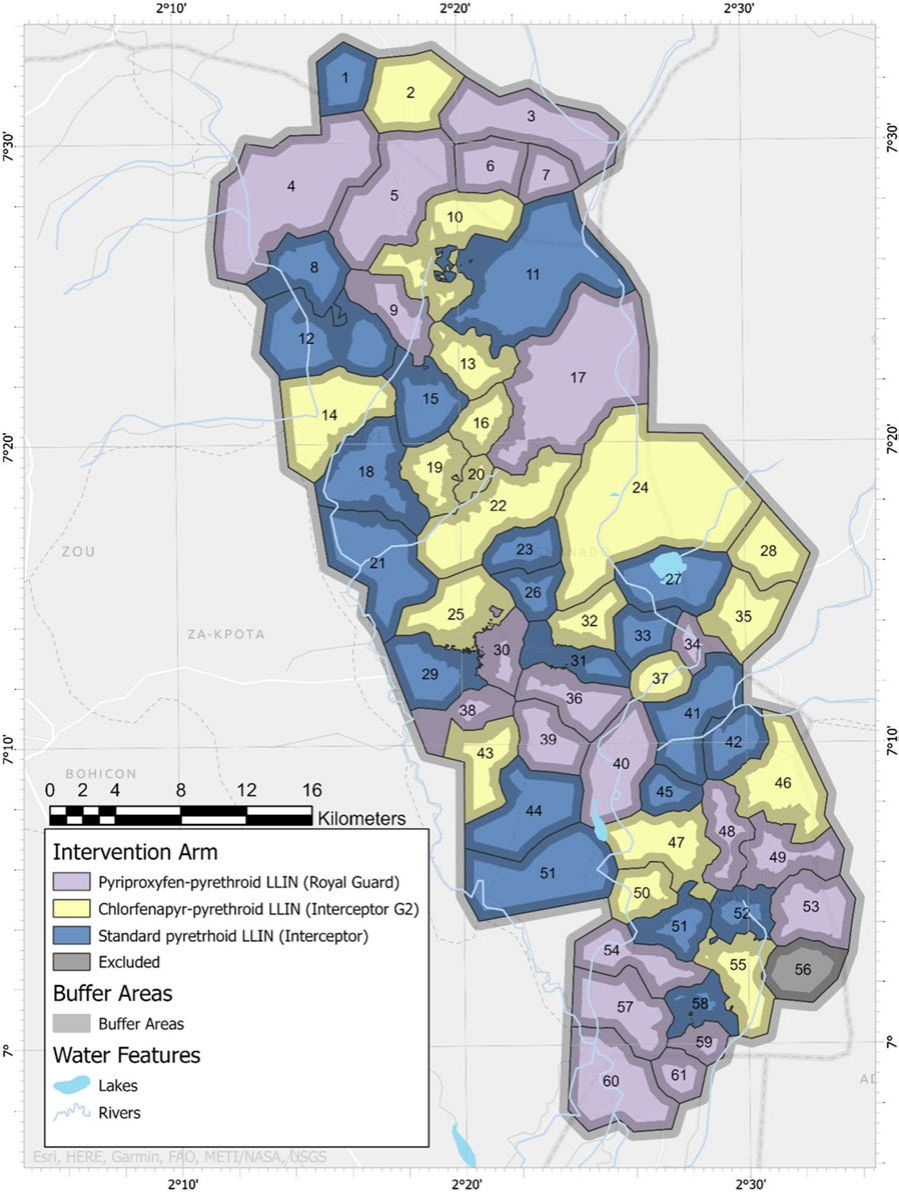
Map of the study area showing the three study arms

The present three-arm cluster RCT was conducted in Cove, Ouinhi and Zagnanado districts, located in the Zou region, Central Benin. Malaria endemicity was high, with transmission occurring year-round. Deployment of LLINs every 3 years remained the principal vector control strategy in this region where *An. coluzzii* and *An. gambiae* s.s., the main malaria vector species, displayed high pyrethroid resistance intensity [12]. The study area and trial design have been described previously [13] and the primary analyses of the trial were also published previously [6]. Briefly, the region consisted of 123 villages divided into 60 clusters, each formed from a village or a group of villages. Restricted randomization was used to randomly assign 20 clusters to each of the three study LLINs (Fig. 1). These were: (i) Royal Guard^®^ LLIN, a 120-denier polyethylene net incorporating a mixture of 220 mg/m^2^ alpha-cypermethrin and 220 mg/m^2^ pyriproxyfen (Disease Control Technologies, Greer, SC, USA), (ii) Interceptor^®^ G2^®^ LLIN, a 100-denier polyester net coated with 200 mg/m^2^ chlorfenapyr and 100 mg/m^2^ alpha-cypermethrin (BASF SE, Ludwigshafen, Germany), and (iii) Interceptor^®^ LLIN, a 100-denier polyester netting that incorporates 200 mg/m^2^ of alpha-cypermethrin (BASF SE, Ludwigshafen, Germany).

### Procedures

**Fig. 2:**
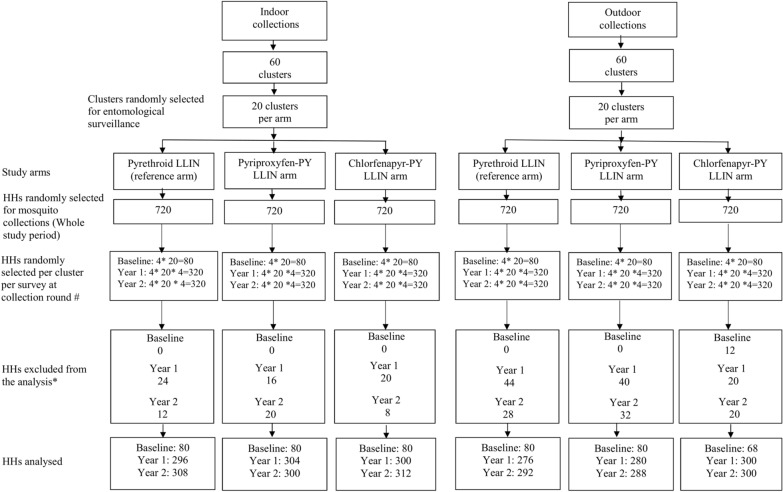
Trial profile for the vector density. *HHs* household visits. *LLIN* long-lasting insecticidal net. *PY* pyrethroid. *PPF* pyriproxyfen. # For each cluster, four households were randomly selected for each collection rounds. *HH excluded from the analysis are those belonging to clusters with: **→** no mosquito speciated while number collected ≥ 1; **→** 1 ≤ number collected ≤ 10 and 0% < % speciated < 30%; **→** number collected > 10 and 0 < number speciated < 5. Four consecutive collection rounds were performed in each of the 2 post-intervention study years

Written consent to participate in the trial was sought from the household heads, and the adult volunteers that collected mosquitoes through human landing catches (HLCs), after being vaccinated against yellow fever. Prior to the study net distribution, one round of mosquito collection occurred between September and October 2019. The net distribution was conducted in March 2020 with support from the National Malaria Control Programme, with a ratio of one net for every two people. Due to the COVID-19 pandemic, there was no data collection between April and May 2020; then entomological collections using HLCs in each cluster were conducted every 3 months leading to eight collection rounds between June 2020 and April 2022 [6–13]. A total of four houses were surveyed in the core area of each of the 60 clusters per round (total 240 collection nights indoors and 240 outdoors per round), with the first randomly selected and the three others chosen in a 20-m radius around the first. In each house, collections were done indoors and outdoors from 19:00 to 7:00. Each night a first group of trained collectors worked between 7:00 p.m. and 01:00 a.m. and were substituted by a second group between 01:00 and 07:00 a.m. They used haemolysis tubes and flashlights to collect all mosquitoes on their lower limbs before they received any bites. Collected mosquitoes were morphologically identified to species level using a binocular microscope and the taxonomic identification key of Gillies et al. [14]. About 15% of *An. gambiae* s.l. randomly sampled across collection hours and locations (indoor and outdoor) were dissected to assess the parity rate [15]. Molecular species identification was also performed using PCR [16]. The trial profile is provided in Fig. 2.

### Outcomes

The primary entomological outcome was measured indoors and outdoors for both *An. gambiae* s.s. and *An. coluzzii.* The density of vectors was defined as the estimated mean number of each mosquito species collected per person per night. This indicator, measured at the cluster-visit level, was calculated at baseline and averaged across collections for year 1 and year 2 post-net distribution. Density was compared between each intervention arm and the pyrethroid LLIN arm (control arm). Secondary entomological outcomes included were mosquito species composition, relative proportion of each molecular species infected and parity rate (the proportion of *An. gambiae* s.l. found parous).

### Statistical analysis

A double entry of the entomological monitoring data was performed in CS Pro 7.2 software-designed databases. The datasets were cleaned with Stata 15.0 (Stata Corp., College Station, TX). As only a proportion (around 15%) of the total *Anopheles* collected were speciated, molecular species density was calculated at cluster level by multiplying the mean number of *An. gambiae* s.l. per cluster visited by the proportion of molecular species (*An. coluzzii* and *An. gambiae* s.s.). Some household visit data were excluded from the analysis using the following criteria:

- No mosquito speciated while number collected ≥ 1,
- 1 ≤ Number of collected mosquitoes ≤ 10, and 0% < % of speciated mosquitoes < 30%,
- Number of collected mosquitoes > 10, and 0 < number of mosquito speciated < 5.

The parity rate per species was calculated by dividing the number of parous mosquitoes by the total mosquitoes dissected.

Vector density, and parity rate were calculated at the cluster level. To analyse the vector density, a mixed-effect generalised linear model with a negative binomial distribution was used, while a mixed-effect logistic regression was used for parity rate. Cluster was included as a random effect.

## Results

### Baseline characteristics of the study area: mosquito species composition, vector density and parity rate

**Fig. 3:**
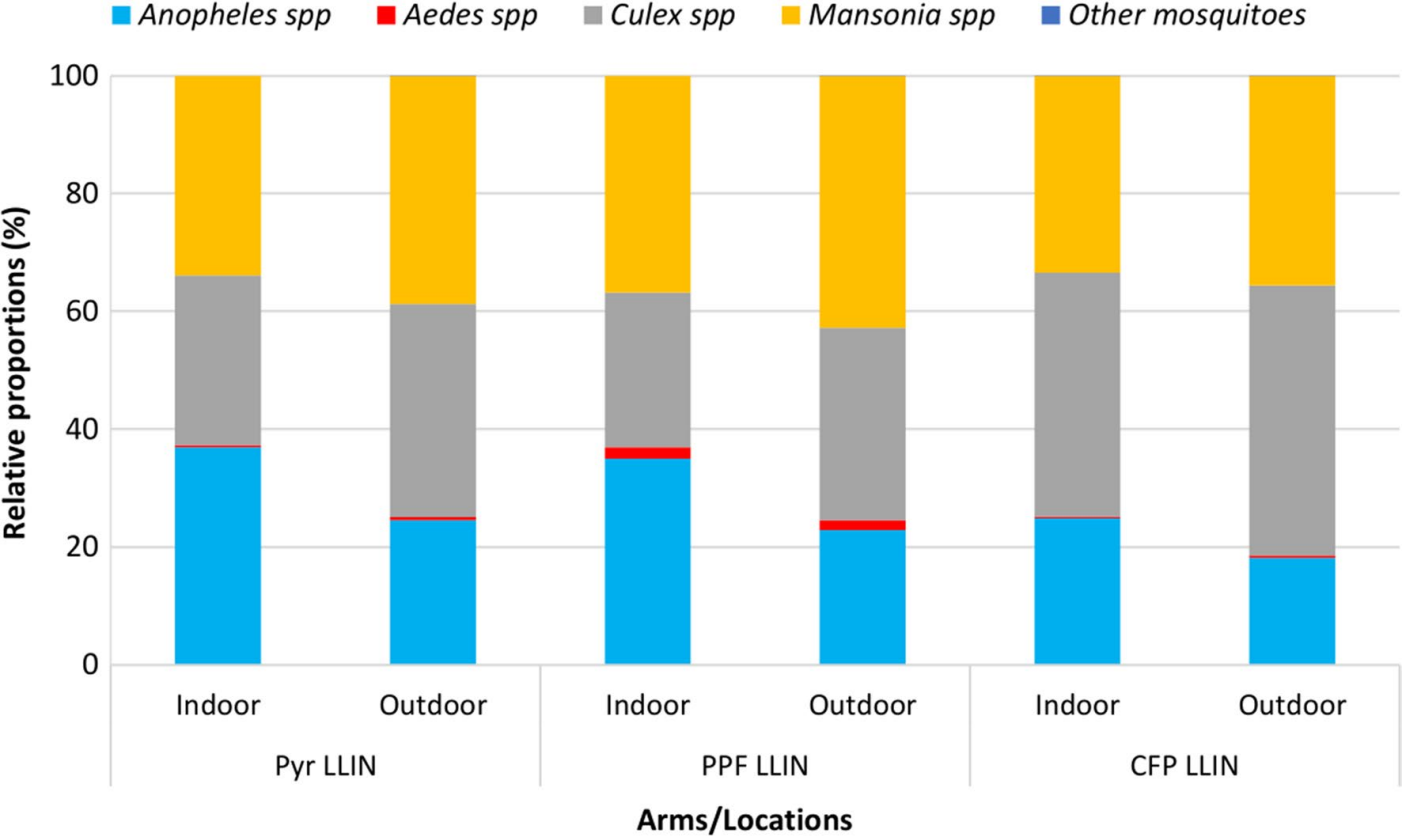
Relative proportions of mosquito species collected indoors and outdoors at baseline in the three study arms. *Pyr LLIN* pyrethroid LLIN arm, *Pyr-PPF LLIN* pyrethroid-pyriproxyfen LLIN arm, *Pyr-CFP LLIN* pyrethroid chlorfenapyr LLIN arm

At baseline, a total of 46,613 mosquito specimens were collected, with 51.6% collected outdoors and the rest indoors. Overall, *Anopheles* mosquitoes accounted for 32.2% (*n* = 7264) of the mosquitoes collected indoors and varied between 24.9 and 37% of the total caught according to trial arm (Fig. 3). The majority of *Anopheles* were *An. gambiae* s.l. (87.7%). *Anopheles* mosquito species found in lower proportions included: *Anopheles ziemanni* (1.5%), followed by *An. pharoensis* (1.2%), *An. funestus* (0.9%) and *An. nili* (0.2%). Other mosquito species found by order of abundance were: *Mansonia* spp. (arm level range: 33.4–36.8%) *Culex* spp. (arm level range: 26.2–41.4%), *Aedes* spp. (arm level range: 0.3–2.0) and *Coquillettidia* spp. and *Eretmapodites* spp. (< 0.1%). Trends were similar indoors and outdoors (Fig. 3).

During this period, a total of 1797 *An. gambiae* s.l. (sporozoite positive samples plus a subset of randomly selected negative ones) were tested by PCR to identify sibling species. Overall, we found 53.9% *An. coluzzii*, and the rest were *An. gambiae* s.s. The relative proportion of *An. coluzzii* was usually the highest in all the arms, indoors and outdoors, except for the pyrethroid-CFP LLIN indoors where *An. gambiae* s.s. was found in the majority (Table 1).

**Table 1:** Baseline characteristics of the study area

Indicators	Study arms	Indoor	Outdoor
Proportion of molecular species in the processed samples	Pyr LLIN: % (95% CI), *N*	54.0 (37.3–70.8), 385	58.1 (38.8–77.5), 215
[(*Anopheles coluzzi*/(*An. coluzzii* + *An. gambiae* s.s)]	Pyr-PPF LLIN: % (95% CI), *N*	55.6 (37.3–74.0), 392	61.2 (43.2–79.2), 201
	Pyr-CFP LLIN: % (95% CI), *N*	43.2 (26.5–59.9), 389	58.6 (42.9–74.3), 215
Estimated density	Pyr LLIN: Mean (95% CI)	14.2 (7.6–20.8)	14.6 (6.4–22.8)
(*An. coluzzii*)	Pyr-PPF LLIN: Mean (95% CI)	17.6 (8.6–26.6)	12.2 (5.6–18.9)
	Pyr-CFP LLIN: Mean (95% CI)	10.6 (4.4–16.8)	10.4 (5.1–15.7)
Estimated density	Pyr LLIN: Mean (95% CI)	10.0 (4.2–15.9)	10.2 (3.7–16.6)
(*An. gambiae s.s.*)	Pyr-PPF LLIN: Mean (95% CI)	11.4 (5.4–17.5)	8.0 (3.6–12.5)
	Pyr-CFP LLIN: Mean (95% CI)	9.3 (4.3–14.4)	6.8 (2.1–11.4)
Parity rate	Pyr LLIN: % (95% CI), *N*	79.9 (74.1–85.7), 563	80.7 (74.7–86.6), 367
(*An. gambiae* s.l.)	Pyr-PPF LLIN: % (95% CI), *N*	81.5 (75.2–87.7), 635	77.6 (69.3–85.8), 325
	Pyr-CFP LLIN: % (95% CI), *N*	85.1 (80.3–89.9), 582	79.8 (72.9–86.8), 371

*An*. *Anopheles*, *N* total number of tested mosquitoes, *Pyr LLIN* pyrethroid LLIN arm, *Pyr-PPF LLIN* pyrethroid-pyriproxyfen LLIN arm, *Pyr-CFP LLIN* pyrethroid chlorfenapyr LLIN arm. The density was estimated at the cluster visit level

The estimated density of *An. coluzzii* ranged between 10.6 and 17.6 bites/person/night (b/p/n) indoors and between 10.4 and 14.6 b/p/n outdoors according to arms. *Anopheles gambiae* s.s. estimated density varied between 9.3 and 11.4 b/p/n indoors and between 6.8 and 10.2 b/p/n outdoors (Table 1).

Overall, 82.1% of the total *An. gambiae* s.l. collected indoors and dissected (1461/1780) were found parous and 81.5% (866/1063) of those collected outdoors. Those proportions were similar among the three study arms (Table 1).

### Post-intervention

#### Mosquito species composition

**Fig. 4:**
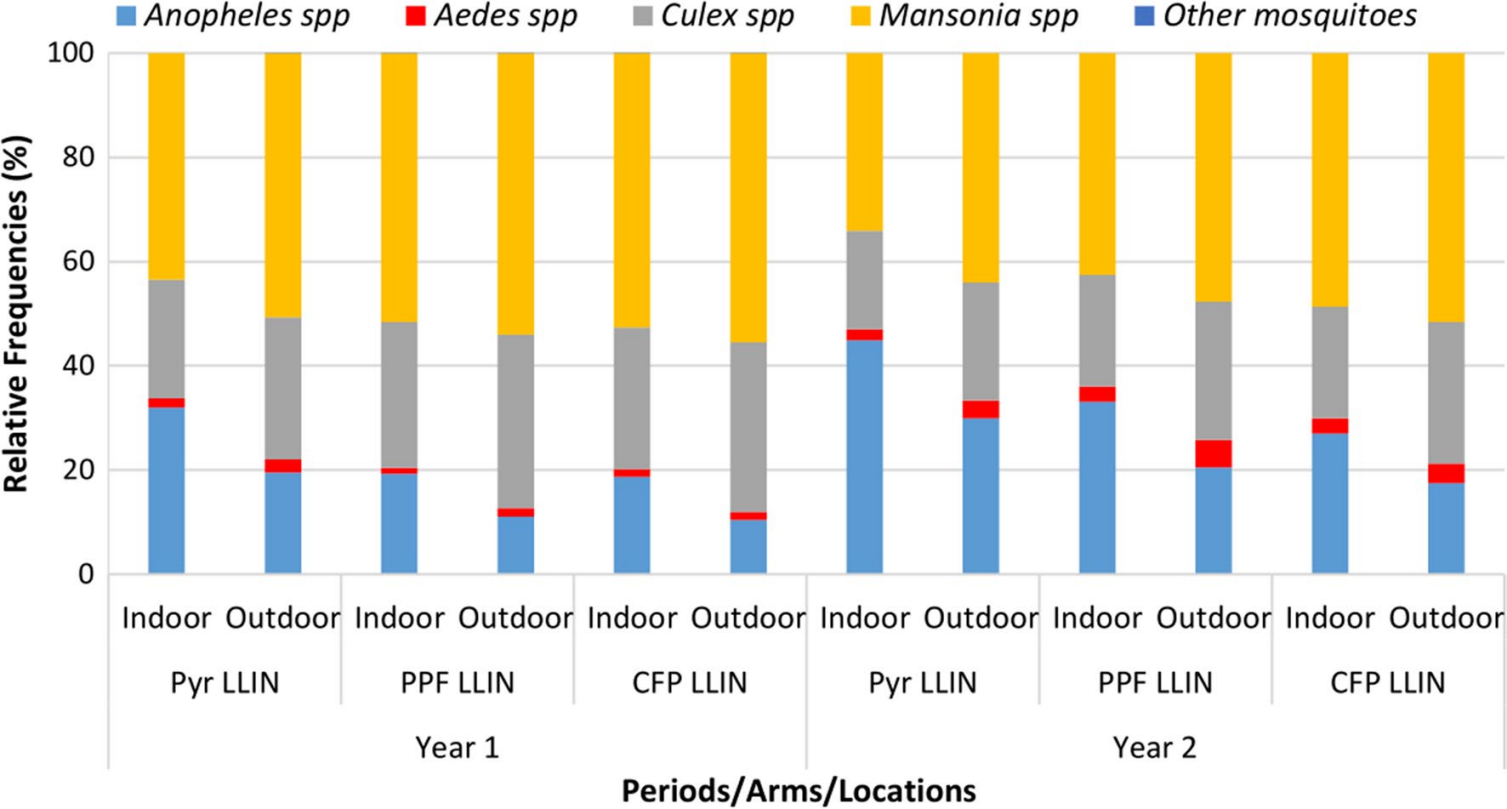
Relative proportions of mosquito species collected indoors and outdoors post-intervention in the three study arms. *Pyr LLIN* pyrethroid LLIN arm, *Pyr-PPF LLIN* pyrethroid-pyriproxyfen LLIN arm, *Pyr-CFP LLIN* pyrethroid chlorfenapyr LLIN arm

In the first year of the trial (between June 2020 and March 2021), a total of 161,569 mosquitoes were collected with a higher density outdoors (58.2%). Overall, the proportion of *Anopheles* collected was 23.6% (15,926/67,497) indoors and 13.8% (15,926/94,072) outdoors, with the lowest proportion found in the pyrethroid-CFP LLIN arm (28.5%) compared to pyrethroid-PPF LLIN arm (37.1%) and pyrethroid-only LLIN arm (34.4%). The proportion of *Culex* spp. caught was lower compared to baseline, whilst *Mansonia* spp. was higher across the study arms. As observed in baseline, *Aedes* spp. (< 3%) and other mosquitoes including both *Coquillettidia* spp. and *Eretmapodites* spp. (≤ 0.02%) were collected at lower proportions (Fig. 4).

In the second year of the trial, a total of 97,681 mosquito specimens were collected between April 2021 and April 2022. Overall, 28.8% (*n* = 28,084) of the total mosquitoes collected were *Anopheles*, with 14,876 sampled indoors and the rest outdoors. Relative proportions of *Anopheles* spp. and *Aedes* spp., both indoors and outdoors, were higher than those observed in year 1. The opposite trend was observed in *Mansonia* spp., and to a lesser extent in *Culex* spp. (Fig. 4).

A total of 8185 of the 53,723 *An. gambiae* s.l. collected both indoors and outdoors were tested for molecular species identification during the 2 years post-intervention. On average, *An. coluzzii* accounted for 71.9% of the *An. gambiae* s.l. indoors and outdoors over the 2 years. Across both years, indoor proportions were similar between arms and ranged between 70.5 and 73.0%, while the outdoor proportions ranged between 69.3 and 73.3% (Table 2). The majority of the remaining mosquitoes were *An. gambiae* s.s. with a small proportion (< 1%) of hybrids (*An. gambiae/coluzzii*) (Table 2). Species composition changed according to season and the relative proportion of *An. coluzzii* increased and peaked during the dry season between December and April each year and was the lowest in September–October during the rainy season (Additional file 1: Figure. S1).

**Table 2:** Relative proportions of *Anopheles coluzzii* and *An. gambiae* s.s. indoors and outdoors, and across study arms

Periods	Arms	Total tested		Indoor				Outdoor	
			*An. gambiae s.s*	*An. coluzzii*	*An. gambiae/coluzzii*		*An. gambiae s.s*	*An. coluzzii*	*An. gambiae/coluzzii*
			% (95% CI), *N*	% (95% CI), *N*	% (95% CI), *N*	Total tested	% (95% CI), *N*	% (95% CI), *N*	% (95% CI), *N*
Year 1:Post intervention	Pyr LLIN	895	32.0 (17.9–46.0), 271	67.7 (53.69–81.8), 621	0.3 (0–0.6), 3	532	34.5 (15.7–53.3), 170	65.0 (46.1–83.9), 359	0.50 (0–1.04), 3
	Pyr-PPF LLIN	690	29.3 (12.5–46.2), 198	70.6 (53.69–87.4), 490	0.1 (0–0.3), 1	545	33.0 (13.2–52.9), 178	66.7 (46.8–86.5), 364	0.30 (0–0.71), 2
	Pyr-CFP LLIN	780	27.6 (15.3–39.9), 214	72.4 (60.03–84.7), 566	0	538	25.5 (13.7–37.2), 137	74.4 (62.6–86.1), 400	0.16 (0–0.46), 1
Year 2:Post intervention	Pyr LLIN	869	22.0 (9.06–34.9), 188	77.4 (64.14–90.7), 675	0.6 (0–1.32), 6	759	24.8 (9.3–40.3), 185	74.6 (59.1–90.1), 569	0.60 (0–1.34), 5
	Pyr-PPF LLIN	738	31.4 (12.14–50.7), 230	68.1 (48.60–87.5), 503	0.5 (0.06–0.95), 4	582	26.2 (7.4–45.0), 150	73.4 (54.7–92.1), 429	0.42 (0–0.87), 3
	Pyr-CFP LLIN	696	24.7 (10.12–39.2), 168	74.5 (59.6–89.4), 521	0.8 (0.16–1.51), 7	561	30.9 (16–45.8), 173	68.8 (53.7–83.9), 386	0.34 (0–0.78), 2
Overall: Post-intervention	Pyr LLIN	1764	26.5 (13.2–39.7), 459	73.0 (59.7–86.4), 1296	0.5 (0.06–0.9), 9	1291	26.2 (11.3–40.98), 355	73.3 (58.3–88.2), 928	0.58 (0.10–1.06), 8
	Pyr-PPF LLIN	1428	29.2 (11.7–46.7), 428	70.5 (52.9–87.9), 993	0.34 (0.16–0.52), 5	1127	30.3 (12.2–48.4), 328	69.3 (51.2–87.4), 793	0.42 (0.18–0.65), 5
	Pyr-CFP LLIN	1476	27.5 (14.1–41.0), 382	72.1 (58.5–85.7), 1087	0.37 (0.08–0.66), 7	1099	26.8 (13.6–40.1), 310	72.7 (59.3–86.2), 786	0.47 (0.14–0.80), 3

An. *Anopheles*, *N* number, *Pyr LLIN*: pyrethroid LLIN arm, *Pyr-PPF LLIN* pyrethroid-pyriproxyfen LLIN arm, *Pyr-CFP LLIN* pyrethroid chlorfenapyr LLIN arm

#### Density in *An. gambiae* s.s. and *An. coluzzii*

Overall (year 1 + year 2), indoor estimated density of *An. coluzzii* in the pyrethroid-only LLIN arm was 18.6 b/p/n compared to 8.1 b/p/n in the pyrethroid-chlorfenapyr LLIN arm (DR = 0.56 95% CI (0.19–1.62); *p* = 0.2866) and 10.4 b/p/n in the pyrethroid-pyriproxyfen LLIN arm (DR = 0.62 95% CI (0.21–1.77); *p* = 0.3683). A non-significant reduction in density was observed in years 1 and 2 post-net distribution (indoors and outdoors) (Table 3).

**Table 3:** Indoor and outdoor density of *Anopheles coluzzii* and *An. gambiae* s.s. across study arms

					*An. coluzzii*			*An. gambiae* s.s		
Locations	Periods	Arms	N of *An. gambiae* s.l	Total	Mean	DR (95% CI)	*p*-value	Mean	DR (95% CI)	*p*-value
				cluster-visits						
Indoor										
	Year 1	Pyr LLIN	6770	296	19.2	1 (Ref)		3.7	1 (Ref)	
		Pyr-PPF LLIN	4386	304	11.8	0.60 (0.20–1.78)	0.3584	2.7	0.49 (0.19–1.27)	0.1422
		Pyr-CFP LLIN	3455	300	9.6	0.69 (0.23–2.05)	0.5054	1.9	0.52 (0.20–1.32)	0.1703
	Year 2	Pyr LLIN	6891	308	17.9	1 (Ref)		4.5	1 (Ref)	
		Pyr-PPF LLIN	3869	300	8.9	0.62 (0.20–1.92)	0.4096	3.9	0.59 (0.16–2.26)	0.4474
		Pyr-CFP LLIN	2642	312	6.6	0.45 (0.14–1.40)	0.1692	1.9	0.49 (0.13–1.83)	0.2902
	Overall	Pyr LLIN	13661	604	18.6	1 (Ref)		4.1	1 (Ref)	
		Pyr-PPF LLIN	8255	604	10.4	0.62 (0.21–1.77)	0.3683	3.3	0.52 (0.18–1.46)	0.2192
		Pyr-CFP LLIN	6097	612	8.1	0.56 (0.19–1.62)	0.2866	1.9	0.53 (0.19–1.46)	0.2222
Outdoor										
	Year 1	Pyr LLIN	5448	276	16.4	1 (Ref)		3.3	1 (Ref)	
		Pyr-PPF LLIN	3107	280	8.8	0.51 (0.16–1.69)	0.2768	2.5	0.52 (0.18–1.51)	0.2333
		Pyr-CFP LLIN	2495	300	6.7	0.58 (0.18–1.91)	0.3784	1.5	0.45 (0.16–1.29)	0.1406
	Year 2	Pyr LLIN	5199	292	14.2	1 (Ref)		3.6	1 (Ref)	
		Pyr-PPF LLIN	3366	288	8.0	0.75 (0.24–2.33)	0.6152	3.7	0.91 (0.23–3.64)	0.8995
		Pyr-CFP LLIN	2448	300	5.7	0.57 (0.18–1.77)	0.3316	2.5	0.99 (0.25–3.90)	0.9921
	Overall	Pyr LLIN	10,647	568	15.3	1 (Ref)		3.5	1 (Ref)	
		Pyr-PPF LLIN	6473	568	8.4	0.71 (0.21–2.37)	0.5792	3.1	0.80 (0.26–2.44)	0.6997
		Pyr-CFP LLIN	4943	600	6.2	0.61 (0.18–2.05)	0.4317	1.9	0.77 (0.25–2.31)	0.6374

*An*. *Anopheles*, *N* number, *Pyr LLIN* pyrethroid LLIN arm, *Pyr-PPF LLIN* pyrethroid-pyriproxyfen LLIN arm, *Pyr-CFP LLIN* pyrethroid chlorfenapyr LLIN arm, *DR* density ratio, The density was estimated at the cluster visit level

*Anopheles gambiae* s.s. estimated indoor density was overall lower in the pyrethroid-pyriproxyfen LLIN arm [3.3 b/p/n, DR = 0.52 95% CI (0.18–1.46); *p* = 0.2192] and the pyrethroid-chlorfenapyr LLIN arm [1.9 b/p/n, DR = 0.53 95% CI (0.19–1.46); *p* = 0.2222] compared to the pyrethroid LLIN arm (4.1 b/p/n); however, this difference was not significant. This was also observed in each of the 2 years post-net distribution. Similar trends were found outdoors (Table 3).

#### Parity rate (PR) in *An. gambiae* s.l.

Overall, there was no evidence of a reduction in the parity rate in the two intervention arms compared to the control arm both indoors [PR = 81.6%, OR = 1.3 (95% CI 0.9–1.8), *p* = 0.2014 in the pyrethroid-pyriproxyfen LLIN arm, and PR = 79.8%, OR = 1.1 (95% CI 0.7–1.5), *p* = 0.7532 in the pyrethroid-chlorfenapyr LLIN arm, versus PR = 78.6% in the pyrethroid LLIN arm] and outdoors [PR = 80.2%, OR = 1.2 (95% CI 0.9–1.6), *p* = 0.2717 in the pyrethroid-pyriproxyfen LLIN arm, and PR = 80.2%, OR = 1.1 (95% CI 0.8–1.5), *p* = 0.5642 in the pyrethroid-chlorfenapyr LLIN arm, versus PR = 78.6% in the pyrethroid LLIN arm] in the first year of the trial. The same trend was observed during the first and the second year of the trial (Table 4).

**Table 4:** Parity rate in *Anopheles gambiae* s.l

		Indoor parity rate						Outdoor parity rate					
Period	Arms	Number parous	Number dissected for parity	%	95% CI	OR (95% CI)	*p*-value	Number parous	Number dissected for parity	%	95% CI	OR (95% CI)	*p*-value
Year 1	Pyr LLIN	1243	1624	77.1	71.3–82.8	1 (Ref)	–	1019	1300	77.0	72.2–81.8	1 (Ref)	–
	PPF LLIN	1058	1333	80.0	73.6–86.4	1.3 (0.8–2.1)	0.2906	955	1189	76.1	68.2–84	1.2 (0.7–1.9)	0.5203
	CFP LLIN	998	1313	78.0	73.3–82.7	1.0 (0.6–1.6)	0.9997	803	1035	79.4	74.8–84.1	1.1 (0.6–1.7)	0.8148
Year 2	Pyr LLIN	1172	1520	78.9	74.8–83	1 (Ref)	–	1026	1329	79.9	74.2–85.6	1 (Ref)	–
	PPF LLIN	1207	1530	80.9	76.6–85.2	1.1 (0.7–1.7)	0.7572	1100	1333	84.1	80.5–87.7	1.4 (0.8–2.4)	0.1671
	CFP LLIN	985	1197	82.5	76.4–88.6	1.3 (0.8–2.1)	0.298	898	1108	78.7	73.6–83.9	1.1 (0.7–1.9)	0.6804
Overall	Pyr LLIN	2415	3144	78.6	75–82.3	1 (Ref)	–	2045	2629	78.6	75.4–81.8	1 (Ref)	–
	PPF LLIN	2265	2863	81.6	78.1–85.1	1.3 (0.9–1.8)	0.2014	2055	2522	80.2	77.1–83.4	1.20 (0.87–1.6)	0.2717
	CFP LLIN	1983	2510	79.8	76.5–83	1.1 (0.7–1.5)	0.7532	1701	2143	80.2	76.7–83.8	1.10 (0.8–1.5)	0.5642

An. *Anopheles*, *N* number, *Pyr LLIN* pyrethroid LLIN arm, *Pyr-PPF LLIN* pyrethroid-pyriproxyfen LLIN arm, *Pyr-CFP LLIN* pyrethroid chlorfenapyr LLIN arm

## Discussion

This secondary analysis provides further insights on the impact of pyrethroid-chlorfenapyr and pyrethroid-pyriproxyfen LLINs on the two main malaria vectors found in the Zou region, Southern Benin. *Anopheles coluzzii* and *An. gambiae* s.s. are commonly found circulating sympatrically across West Africa, but differ in their larval ecology, behaviour, migration, aestivation, and insecticide resistance mechanisms [17–22]. There is some indication that the impact of pyrethroid-chlorfenapyr LLIN was similar on both species (*An. gambiae* s.s. and *An. coluzzii*) with a slight reduction in year 2 on *An. gambiae* s.s. especially outdoors. Similar observation was found with pyrethroid-pyriproxyfen LLINs with some indication that the effect might be less than for the pyrethroid-chlorfenapyr LLIN.

One of the key factors for the acceptance of a vector control tool by a community is its ability to reduce the mosquito biting frequency. In the present trial, though there was not strong evidence, both pyrethroid-chlorfenapyr LLINs and pyrethroid-pyriproxyfen LLINs were found to reduce the density of *An. coluzzii* and *An. gambiae* s.s. at a broadly similar magnitude, both indoors and outdoors. By comparison, a clear differential effect was observed between the two LLINs after aggregating data of the three main malaria vector complexes (*An. gambiae* s.l. + *An. funestus* + *An. nili*) encountered in the study area, as the pyrethroid-pyriproxyfen LLINs reduced the indoor vector density by 42% (*p* = 0.11), while the pyrethroid-chlorfenapyr LLINs did significantly by 56% (*p* = 0.014) over the 2 first years of the trial [6]. The same trend was also observed with *An. funestus*, with the chlorfenapyr-pyrethroid LLIN controlling this vector species over 3 years, while the two dual a.i. LLINs had no impact on density of *An. arabiensis* in Tanzania [9]. The weak evidence (*p* > 0.05) for the reductions induced by the two dual a.i. LLINs on the density of the two primary vectors in the present trial could be partly due to data scarcity. The density was estimated at the cluster-visit level rather than at a household level to reduce bias from estimating proportions in small samples. This will have resulted in less power. In addition, from our observations in the field, the pyrethroid-pyriproxyfen LLINs were frequently used outside households for other purposes (fishing, plant protection) given its high shrinkage observed in the field as well as its ability to tear quickly. These factors may have limited the exposure time of vectors to the intervention tool, resulting in reduced sterilization effects of pyriproxyfen on vectors and a lack of effectiveness of this LLIN. Similarly, a trial previously conducted in Burkina Faso also revealed that a pyrethroid-pyriproxyfen LLIN successfully halved the entomological inoculation rate (EIR) but induced a weak reduction in clinical malaria incidence of 12% [23]. When comparing the indoor and outdoor impact of the two dual ai-LLINs, it appeared to have a greater effect indoors than outdoors, thus emphasizing the need for outdoor complementary vector control tools. Furthermore, the broadly similar impact that each of the two dual a.i. LLINs tended to have on the density of *An. gambiae* s.s. and *An. coluzzii* suggests that combining these insecticides (chlorfenapyr and pyriproxyfen) with the pyrethroid insecticide (alpha-cypermethrin) in the LLINs had a similar effect on the density of the two primary vectors.

Over the 2 years after the net distribution, about three quarters of the collected specimens of *An. gambiae* s.l. was *An. coluzzii*, while the rest was composed of *An. gambiae* s.s. with a small proportion (< 1%) of hybrids (*An. gambiae*/*coluzzii*). By comparison, the two predominant molecular species were previously found in similar proportions at baseline (50.9% for *An. coluzzii* vs. 49.1% for *An. gambiae* s.s.) [12]. The changes observed in proportions of these two primary species between the post-net distribution period and baseline could be due to the seasonality and/or the differential selection induced by the interventions. Indeed, the baseline collection occurred during only one round performed over the short rainy season (September–October 2019) so could not provide a representative image of the molecular species composition compared to the four rounds of collection (covering all seasons) of each of the two post-net distribution years. Furthermore, during the whole study period, *An. coluzzii* was found to peak over the dry seasons, which corroborates previous works by Salako et al*.* [24] in the northern regions of Atacora and Donga in Benin. This could be because, during that period of the year, there were many permanent/semi-permanent breeding sites created by rice paddies as well as tributaries of the Oueme and Zou rivers that irrigate the study area, the temporary breeding sites being only found during the rainy season. Indeed, according to Diabate et al. [25], permanent/semi-permanent and temporary breeding sites were conducive to the emergence of *An. coluzzii* and *An. gambiae* s.s., respectively.

Limitations of the present study include the lack of data on both entomological inoculation rate and rainfall, which influence vector density.

After dissecting a subsample of *An. gambiae* s.l., the parity rate, which shows the physiological age of mosquito populations, was similar across the three study arms, suggesting that this malaria vector complex has passed through approximately the same number of gonotrophic cycles in the three study arms. This finding corroborates previous results from Accrombessi et al. [6], who showed similar sporozoite rates across the three study arms. This conflicting trend might be due to the fact that, apart from the interventions deployed, parity rates could have been strongly influenced by other factors such as climate conditions (temperature, relative humidity), which can vary from place to place and over time, as previously mentioned by Adugna et al. [26], whoch we did not account for in our analysis. Thus, in a trial evaluating the efficacy of vector control tools, data on parity rates should be interpreted cautiously, given the existence of confounding factors.

## Conclusions

The lack of a significant reduction in the density of primary vectors by either of the two dual active-ingredients LLINs could be because of the low sample size of mosquito speciated. Thus, both pyrethroid-chlorfenapyr LLINs and pyrethroid-pyriproxyfen LLINs appeared to have a similar impact on *An. coluzzii* and *An. gambiae* s.s. in this study.

### Supplementary Information

**Additional file 1: Figure S1.** Seasonal variation of proportion of *Anopheles coluzzii *indoors and outdoors in the study area. Rs: rainy season, Ds: Dry season.

## Data Availability

The data supporting the findings of the study must be available within the article and/or its supplementary materials, or deposited in a publicly available database.
