# Supplementary figures and images for: The impact of pyrethroid-pyriproxyfen and pyrethroid-chlorfenapyr long-lasting insecticidal nets on density of primary malaria vectors Anopheles gambiae s.s. and Anopheles coluzzii in Benin: a secondary analysis of a cluster randomised controlled trial
Source: Parasit Vectors. 2024 Jan 4;17:7. doi: 10.1186/s13071-023-06104-5 (PMC10768265; doi:10.1186/s13071-023-06104-5)

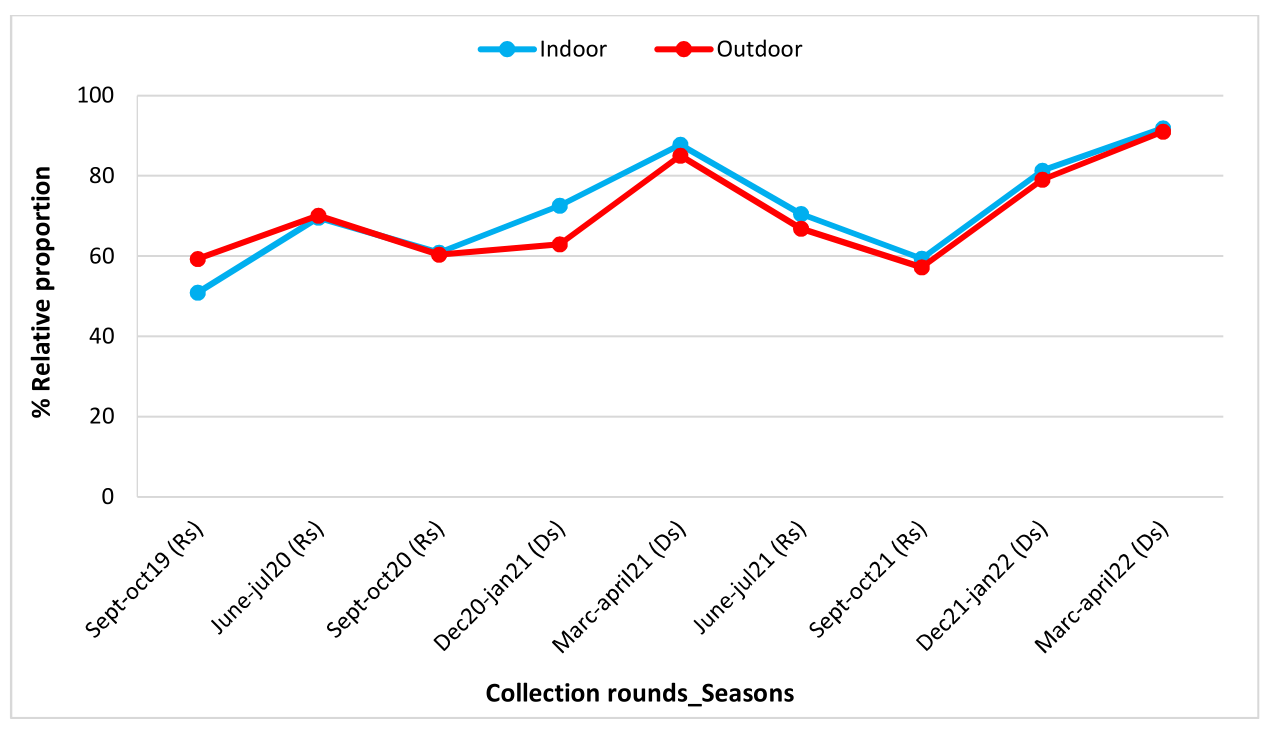

Supplement: Supplementary file 1 — Additional file 1: Figure S1. Seasonal variation of proportion of Anopheles coluzzii indoors and outdoors in the study area. Rs: rainy season, Ds: Dry season. [file 13071_2023_6104_MOESM1_ESM.tif]
